# Ventriculoperitoneal shunt insertion in human immunodeficiency virus infected adults: a systematic review and meta-analysis

**DOI:** 10.1186/s12883-020-01713-4

**Published:** 2020-04-17

**Authors:** James J. M. Loan, Michael T. C. Poon, Steven Tominey, Ncedile Mankahla, Graeme Meintjes, A. Graham Fieggen

**Affiliations:** 1 Centre for Clinical Brain Sciences and Centre for Discovery Brain Sciences, Chancellor’s Building, 49 Little France Crescent, Edinburgh, EH16 4SB UK; 2 Edinburgh Medical School, Chancellor’s Building, 49 Little France Crescent, Edinburgh, EH16 4SB UK; 3 Division of Neurosurgery, University of Cape Town, H53 Old Main Building, Groote Schuur Hospital, Main Road, Observatory, Cape Town, 7925 South Africa; 4 Institute of Infectious Disease and Molecular Medicine, Faculty of Health Sciences, University of Cape Town, Anzio Road, Observatory, Cape Town, 7925 South Africa

**Keywords:** Systematic review, Meta-analysis, Ventriculoperitoneal shunt, Cerebrospinal fluid, Hydrocephalus, Human immunodeficiency virus, Acquired immunodeficiency syndrome

## Abstract

**Background:**

Hydrocephalus is a common, life threatening complication of human immunodeficiency virus (HIV)-related central nervous system opportunistic infection which can be treated by insertion of a ventriculoperitoneal shunt (VPS). In HIV-infected patients there is concern that VPS might be associated with unacceptably high mortality. To identify prognostic indicators, we aimed to compare survival and clinical outcome following VPS placement between all studied causes of hydrocephalus in HIV infected patients.

**Methods:**

The following electronic databases were searched: The Cochrane Central Register of Controlled Trials, MEDLINE (PubMed), EMBASE, CINAHL Plus, LILACS, Research Registry, the metaRegister of Controlled Trials, ClinicalTrials.gov, African Journals Online, and the OpenGrey database. We included observational studies of HIV-infected patients treated with VPS which reported of survival or clinical outcome. Data was extracted using standardised proformas. Risk of bias was assessed using validated domain-based tools.

**Results:**

Seven Hunderd twenty-three unique study records were screened. Nine observational studies were included. Three included a total of 75 patients with tuberculous meningitis (TBM) and six included a total of 49 patients with cryptococcal meningitis (CM). All of the CM and two of the TBM studies were of weak quality. One of the TBM studies was of moderate quality. One-month mortality ranged from 62.5–100% for CM and 33.3–61.9% for TBM. These pooled data were of low to very-low quality and was inadequate to support meta-analysis between aetiologies. Pooling of results from two studies with a total of 77 participants indicated that HIV-infected patients with TBM had higher risk of one-month mortality compared with HIV non-infected controls (odds ratio 3.03; 95% confidence-interval 1.13–8.12; *p* = 0.03).

**Conclusions:**

The evidence base is currently inadequate to inform prognostication in VPS insertion in HIV-infected patients. A population-based prospective cohort study is required to address this, in the first instance.

## Background

### Description of the condition

Human immunodeficiency virus (HIV) is the causative pathogen for the acquired immunodeficiency syndrome (AIDS). Without treatment, AIDS will usually occur within 10 years of HIV seroconversion [1–3]. AIDS may predispose to raised cerebrospinal fluid (CSF) pressures and hydrocephalus by various aetiologies. These include tuberculous meningitis (TBM), cryptococcal meningitis (CM), primary central nervous system (CNS) lymphoma, coccoidal meningitis and immune reconstitution inflammatory syndrome (IRIS) [4–8].

In the absence of permanent CSF diversion therapy, the identification and treatment underlying causative factors will often result in normalisation of CSF hydrodynamics. Sometimes however, a state of chronically raised CSF pressure develops [9]. Age-related cerebral tissue loss as well as HIV associated neurocognitive disorder are associated with enlargement of CSF spaces due to generalised brain atrophy and may result in the radiological appearance of hydrocephalus, but are associated with normal CSF pressure and flow, so called “hydrocephalus ex vacuo” [10, 11]. This is not an indication for surgical treatment and, consequently, is not discussed in this study [10].

Meningeal infections including TBM, cryptococcal meningitis and bacterial meningitis, may impair CSF resorption at arachnoid granulations, resulting in communicating hydrocephalus [9, 12, 13]. Additionally, in TBM, where thick basal exudate may occlude ventricular outflow pathways, non-communicating hydrocephalus can develop [14]. Without appropriate antimicrobial treatment, TBM with associated hydrocephalus is almost universally fatal, although this is believed to be principally a consequence of basal cerebral vasculitis and infarction, rather than hydrocephalus [15, 16]. Even with optimal medical management, death occurs in 30% of HIV non-infected patients with TBM, and many of those in whom antimicrobial management is successful suffer from chronic hydrocephalus [17, 18]. In the context of HIV infection, medically managed TBM has been reported to be associated with over 4 times higher mortality than HIV non-infected patients [18–20]. This excess mortality might be a consequence of synergistic virulence mechanisms of HIV and *Mycobacterium tuberculosis*, depletion of physiological reserve consequent to chronic HIV infection and demographic differences between HIV-infected and non-HIV infected populations [21–26].

Other HIV-associated meningeal infections, such as CM more typically result in communicating hydrocephalus, which usually resolves with successful antimicrobial treatment and intermittent therapeutic lumbar puncture to treat initially raised CSF pressures [9, 13, 27]. However, insertion of a temporary CSF drainage system might be required to acutely control extremely high initial CSF pressures and, where raised pressure persists, a permanent CSF shunt may be required [28].

### Description of the intervention

A ventriculoperitoneal shunt (VPS) consists of a surgically implanted catheter running from the cerebral ventricles into the peritoneal cavity. This permits drainage of excess CSF according to prespecified parameters determined by a pressure or flow-regulated valve [29]. To reduce VPS associated infection, the catheter may be impregnated with the antibiotics rifampicin and clindamycin [30]. Catheter impregnation with silver has also been trialled [30].

Complications of VPS insertion include infection, catheter occlusion, migration or fracture, and over- or under-drainage [31]. In a United States adult cohort, approximately 20% of patients suffered a complication in the first year post-insertion, whereas in a Kenyan, mostly paediatric, cohort this rate was recorded at 35% [32, 33]. This latter cohort possibly underestimates complication rates in this high-HIV prevalence, lower-middle income country because of high risk of attrition bias [33–35].

Alternatives to VPS include ventriculoatrial and ventriculopleural shunting. Where acute, temporary cranial CSF diversion is required an external ventricular drain (EVD) may be sited as a primary procedure [29]. For some patients with communicating hydrocephalus it may be possible to avoid cranial surgery by draining CSF through serial lumbar puncture, a lumbar drain or a lumboperitoneal shunt [29]. Endoscopic third ventriculostomy may be used to treat some patients with non-communicating hydrocephalus, but is skill and resource intensive and may not be available in emergency or resource limited settings [14].

### Why it was important to conduct this review

HIV-infected patients are at high risk of developing conditions which predispose to hydrocephalus, such as TBM or CM [36, 37]. However, VPS insertion which is a mainstay of treatment for hydrocephalus in HIV non-infected individuals might be particularly high risk in the context of HIV: HIV infection might predispose to shunt infection and patients with hydrocephalus in the context of advanced HIV infection, might have such poor overall predicted survival as to render neurosurgical intervention futile or inappropriate [20, 38]. The literature from HIV non-infected patient populations therefore cannot be generalised to those with HIV infection. Clinicians therefore require a dedicated synthesis of high-quality evidence to appropriately counsel patients and their relatives of the risks and benefits of VPS insertion in the context of HIV.

### Aims and hypotheses

We aimed to identify all published studies of survival and outcome in HIV-infected patients undergoing VPS using a systematic review process and synthesise these data using quantitative and narrative meta-analyses to determine the risks and benefits of VPS insertion in the context of HIV infection. We hypothesised that the evidence base would be insufficient to reliably inform clinical practice or to conduct quantitative meta-analysis.

### Objectives

Our objectives were to: 1) Compare post-VPS mortality at 12, 6 and 1 months between the different aetiologies of HIV-associated hydrocephalus; 2) Produce a narrative review of risks of VPS infection and malfunction and other complications in HIV-infected patients and; 3) Identify baseline patient characteristics predictive of good outcome following VPS in HIV infection.

## Methods

### Study design and protocol registration

We conducted a systematic review of all published literature stored in 10 electronic online databases, with no language or date restrictions. Our study protocol has been published in a peer-reviewed open access format and is registered on PROSPERO (CRD42016052239) [39, 40]. The methods used are summarised here.

### Eligibility criteria

To ensure that no relevant studies were arbitrarily excluded from this work, we included all studies which met all of the below criteria:

### Types of studies

Reports of randomised or non-randomised studies, excepting < 2-person case series, were eligible for inclusion.

### Types of participants

We included studies of HIV-infected patients aged 16 years or older who underwent a VPS procedure for any indication. This included infectious and non-infectious illness as well as illness unrelated to HIV status.

### Types of interventions

We included studies which described VPS insertion using any technique, catheter or valve.

### Types of outcome measures

We included studied which reported overall survival, AIDS specific mortality, VPS survival or measures of functional outcome/disability. We defined VPS survival as the proportion of patients who have a VPS which is not causing symptoms and has not become infected or undergone surgical revision or removal at any point.

### Outcomes

Our primary outcome was one-year survival post-VPS insertion with comparison between all identified aetiologies of hydrocephalus. Secondary outcomes were overall survival, AIDS specific mortality, VPS survival, risk of perioperative complications and clinical outcome score [41–43] at 1 month, 6 months and one-year post-VPS insertion.

### Search methods for the identification of studies

#### Electronic searches

The following electronic online databases were systematically searched using terms for ventriculoperitoneal shunting or CSF diversion and HIV infection (see supplementary materials for full search strategies): The Cochrane Central Register of Controlled Trials (CENTRAL), MEDLINE (PubMed), EMBASE, CINAHL Plus (EBSCOhost), LILACS (BIREME), Research Registry (www.researchregistry.com), the metaRegister of Controlled Trials (mRCT) (www.controlled-trials.com), ClinicalTrials.gov (www.clinicaltrials.gov) and African Journals Online (AJOL). Grey Literature searching will be performed using the OpenGREY database. These were last updated on 17 October 2019. Our search sensitivity was validated confirming that candidate studies identified during scoping searches are included in the search yields [20, 44–46].

#### Searching other resources

The reference lists of included studies were scrutinized to identify any additional studies for inclusion. Two experts in relevant fields (GM and GF) were consulted to identify key studies not identified by electronic searches. No hand searching was employed.

### Data collection and analysis

#### Selection of studies

Each abstract was independently screened against eligibility criteria by two of three reviewers (JL, NM, MP, ST). Where eligibility criteria were met, or possibly met, the full manuscript was obtained and reviewed by two reviewers to determine final inclusion. Any disagreements were resolved by consensus discussion.

#### Data extraction and management

Data was extracted using standardised proformas (supplementary materials) and entered into Review Manager [47]. Reference management was done using EndNote [48].

#### Assessment of risk of bias in included studies

Each included study was initially assessed independently for risk of bias by two reviewers (JL, MP, ST) using the Canadian National Collaborating Centre for Methods and Tools domain based “Quality Assessment Tool for Quantitative Studies” [49, 50]. Disagreement was resolved by consensus discussion.

#### Measures of effect

Our narrative review summarises each included study’s reported measures of effect [39]. For quantitative meta-analysis, we report pooled odds ratios [51]*.*

#### Dealing with missing data

The corresponding authors of all published abstracts of studies that were potentially eligible for inclusion, but for which a full study report was not published, were contacted electronically to request data not provided by the abstract. All authors contacted were allowed a minimum of 18 months to respond, prior to submission of our completed article.

#### Assessment of heterogeneity

On the basis of extracted data, included studies were compared for clinical and methodological heterogeneity. Clinically and methodologically studies reporting similar outcome measures at similar time points, were assessed for statistical heterogeneity by calculation of the I^2^ statistic (%) [47, 51]. We defined substantial heterogeneity as I^2^ greater than 50%.

### Data synthesis

Included studies were synthesised in a narrative review addressing each of our pre-specified outcomes in turn. The strength of the evidence available to inform each outcome was assessed using the GRADE method and summarised in summary of finding (SOF) tables using GRADEpro [52]. Where substantial clinical, methodological or statistical heterogeneity was detected meta-analysis was not attempted. Comparative meta-analysis of homogenous studies was planned using the Peto fixed-effects method [40]. However, as none of the studies were of high quality, study size varied, and as between-studies variation in control matching was significant, it was not clear that fixed-effect assumptions were met. We therefore elected to use the Mantel-Haenszel method, with random effects, and Review Manager v5.3 [47]. As effect sizes from just two studies could be determined it was not possible to generate meaningful funnel plots. As criteria for further meta-analysis were not met, only limited meta-analysis was conducted. Our planned extended meta-analysis has been detailed previously [39].

### Availability of data

Lists of citations for each stage of review and risk of bias assessment tables are available from the corresponding author.

## Results

### Description of studies

**Fig. 1:**
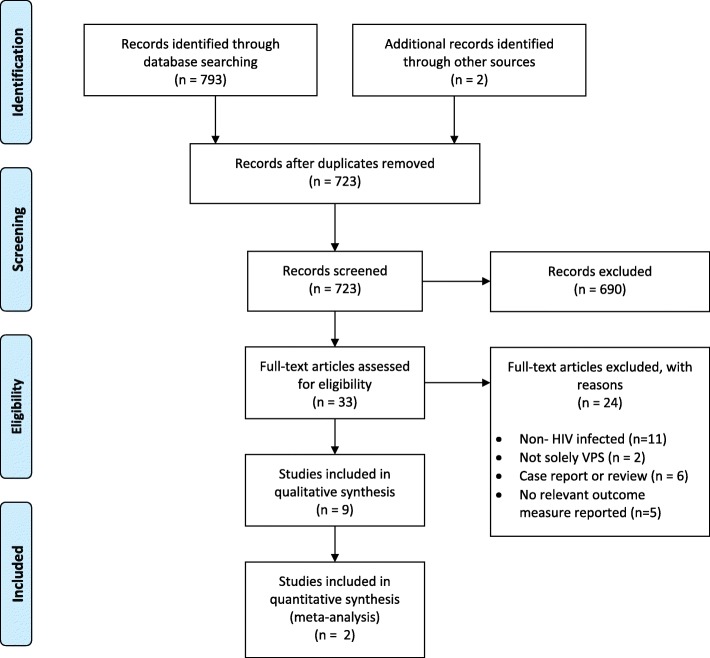
PRISMA Flow diagram of screened and included study reports

Our literature search yielded 793 reports of studies, 721 of which were unique. Two further reports were identified by review of reference lists of included studies and were considered for inclusion [53, 54]. yielding a total of 723 unique records for screening (Fig. 1). No further studies were identified by expert opinion. 624 records were excluded by title and abstract screening. 33 records were selected for full text review [8, 20, 44–46, 53–78]. Nine studies met criteria for inclusion [44–46, 53, 72–75, 79] and 24 were excluded [8, 20, 54–71, 80]. All included studies were observational studies. Three studies included a total of 75 patients with TBM [44, 46, 79] and six included a total of 49 patients with CM [45, 53, 72–75].

#### Excluded studies

Of the excluded studies, 16 concerned CM [54–57, 59–63, 66–69, 76, 78, 80], four TBM [20, 58, 64, 77], two bacterial meningitis [70, 71], one coccoidal meningitis [8], and one included only cases of diagnostic uncertainty [65].

Two studies were published only in abstract form and did not present sufficient outcome data for inclusion [67, 68]. The corresponding authors of these records were therefore contacted to request further details to assess eligibility [8, 67, 68]. The authors one of these responded but were unable to provide further information [8, 68]. This study was therefore excluded.

The reasons for exclusion from this review are as follows: Two studies included patients with multiple, undocumented modalities of CSF diversion [54, 55]; one study provided a just single case report [56]; three studies reported just one or two cases of surgical intervention and no survival/outcome data [57, 60, 61]; one study included 44 patients who underwent VPS in the context of CM but no outcome data [80]; two studies were review articles [58, 66]; eight reported no HIV-infected patients who underwent VPS [20, 59, 62–65, 71, 76–78]; in one study reporting < 50% HIV-infected patients it was not possible to associate outcome with HIV status [8] and; no relevant measures of survival/outcome were studied in four [67–70].

### Studies of cryptococcal meningitis: characteristics and risk of bias

As no comparative studies were included in this group it was impossible to calculate size of effect. Characteristics of included studies are summarised in Table 1 and risk of bias in Table 2.

**Table 1:** Characteristics of included studies

	Aetiology	Number of patients with HIV and VPS	Mean age (years) (SD)^a^	Mean CD4 count (cells/μL; ±SD)^a^	Included outcome	Follow-up^b^	Number of non-HIV infected controls (mean age [±SD])	Country
**Bach 1997** [45]	CM	4	39.25 (±6.1)	28.75(±14.4)	Survival	3d-12y	N/A	USA
**Calvo 2003** [75]	CM	5	28.2 (±7.7)	unknown	Survival	6 m	N/A	Uruguay
**Cherian 2016** [74]	CM	8	38.9 (±8.2)	Unknown	Survival	6 m-5.7y	N/A	USA
**Corti 2014** [73]	CM	14	33 (18–53)*	50 (6–511)*	Survival	1y	N/A	Argentina
**Liu 2014** [72]	CM	9	32.2(±6.0)	10.75(±9.4)	Survival	1 m-1y	N/A	China
**Vidal 2012** [53]	CM	9	Unknown	Unknown	Survival	Discharge	N/A	Brazil
**Nadvi 2000** [46]	TBM	15	26.1 (±16.4)	171.7(±161.5)	GOS	1 m	15 patients (age 10.7[±9.6])	South Africa
**Sharma 2015** [44]	TBM	30	31.3(±7.8)	143 (26–445)**	GOS	3 m	30 patients (age 31[±9.9])	India
**Harrichandparsad 2019** [79]	TBM	30	28.4(±13.4)	227(±163.9)	GOS	1 m	N/A	South Africa

*median (range); ** mean (range); a) HIV infected cohort, b) d = days, m = months, y = years

**Table 2:**
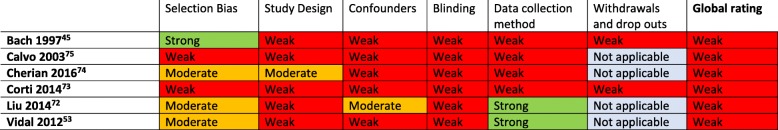
Quality assessment summary for cryptococcal meningitis studies

Using the Canadian national collaborating centre for methods and tools effective public health practice project Quality Assessment Tool for Quantitative Studies [49]

#### Bach, et al. 1997 [45]

This was a retrospective consecutive interrupted time series of all patients admitted with severe intracranial hypertension (lumbar puncture opening pressure > 35cmCSF) secondary to CM who received CSF diversion. Four patients received a VP shunt. Three patients were initially managed with serial lumbar puncture. One received primary VPS. All patients presented with visual disturbance, one had seizures and one had bilateral abducens nerve palsies. Initial Glasgow Coma Score (GCS) was not reported. Neither makes nor models of the VPS systems used were described.

One patient was lost to follow-up at an unclear time point. At 1 month, all three remaining patients were alive. One death at 6 months was attributed to “wasting syndrome”. At 10 months a further death was attributed to *Pneumocystis jiroveci* pneumonia. Functional outcomes, rates of shunt failure and complication were not presented.

Although this study included consecutive patients and therefore minimised selection bias, it was classified as being globally weak. An interrupted time series is a weak study design. Coupled with small sample size, this rendered meaningful multivariable analysis impossible and it was not attempted. The time until loss to follow up of one patient was unclear, and the reasons for this are not documented. Mortality is a robust outcome measure.

#### Calvo, et al. 2003 [75]

This is an interrupted time series that presents five HIV-infected patients who were treated with VPS whilst admitted to a single Intensive Care Unit (ICU) in Montevideo, Uruguay. It is unclear if the data was collected in a prospective or retrospective fashion or whether patients were recruited consecutively. Median initial GCS was 12 (range 9–15). CD4+ cell count was not reported for any patient, nor was the make or model of VPS system. The VPS was inserted as a primary procedure for four patients with CSF pressures >23cmH_2_O. One patient initially received an EVD with intraventricular pressure monitoring before having a VPS inserted. All patients were managed with antifungal agents and hyperventilation to a target pCO_2_ of 30 mmHg. Mannitol boluses were used in an unclear number of patients. At discharge from ICU all five patients were alive. Time to discharge from ICU was not reported. At 6 months two patients had been lost to follow-up and three survived. Outcome in this study is described as “good recovery”, “moderately disabled” or “dead” but the criteria for assignment to these descriptors are not described. At ICU discharge, 4 patients had made a “good recovery” and one was “moderately disabled”. At 3 months, two had a “good recovery” and one was “moderately disabled”. No complications or shunt failure are reported.

This study was classified as being globally weak with a high risk of selection bias and weak study design which, coupled with small sample size, did not permit adjustment for modulators of risk of mortality or poor outcome. Outcome assessment did not use a validated outcome measure. 40% of patients were lost to follow-up at 6 months.

#### Cherian, et al. 2016 [74]

This two-cohort analysis reported all patients with CM and CM IRIS presenting to a single hospital in Texas, USA. 49 of 50 patients had associated HIV infection. Fourty patients had communicating hydrocephalus, and it is unclear if the patient with non-communicating hydrocephalus was HIV-infected or not. CD4+ cell count was not documented for any patient, nor was initial neurological status. All further demographic and outcome data are specific for the HIV-infected group. Patients who went on to receive CSF diversion had initial lumbar puncture opening pressure > 25 cm H_2_O. They were initially managed with antifungals, antiretroviral therapy and a variable number of therapeutic lumbar punctures (median 7; range 2–40) for a variable period (median 27 days; range 4–281 days) before proceeding to VPS. Eight patients underwent VPS.

At 1 month, five of the eight VPS patients survived. Three deaths had occurred, one due to post-extubation aspiration pneumonia, one due to septic shock of undocumented aetiology and the other due to a gram-negative bacterial pneumonia. At 6 months, five patients were still alive, and no new deaths were recorded. At 12 months, three patients were alive, four were dead and one was lost to follow-up. The additional death was secondary to an unspecified respiratory complication. Of the three patients initially treated for CM IRIS, at both one and 6 months, two were alive and one dead. At 12 months, two were confirmed dead and the other lost to follow-up.

One instance of shunt infection was recorded at 12 months. Two other patients, who were followed up for 1512 and 1485 days, suffered a shunt occlusion and a shunt infection, respectively, at some point during their follow-up period. However, the time to these complications was not reported. One other patient who was followed up for 383 days suffered a subdural haematoma, which was attributed to VPS over-drainage. Again, the time point at which this occurred was not reported. Functional outcomes were not reported.

This study was classified as being globally weak with no attempt to undertake multivariable survival analysis, undocumented reasons for loss to follow-up and lack of reporting of times of complications. Survival is a validated outcome measure but was unreliably reported in patients discharged to hospice care. Rates of complication were not measured using a validated method. Patients were consecutively recruited, and the selection process was clearly documented. A two-cohort analysis study has moderate risk of bias.

#### Corti, et al. 2014 [73]

This retrospective interrupted time series included 15 patients with CM who presented to medical services in Buenos Aires, Argentina. 14 of these patients were HIV-infected, however it was not possible to identify which demographic or outcome data pertained to the HIV-non-infected patient. CSF diversion was undertaken in patients who had lumbar puncture opening pressure persistently > 25 cm H_2_0 after 2 weeks of therapeutic lumbar puncture and antifungal treatment. 14 of the 15 patients were managed with VP shunt, and one with lumboperitoneal shunting. Again, it was not possible to isolate demographic and outcome data from this patient. Medtronic® systems were used for all patients but component models are not described.

Three patients suffered complications including sepsis, multiorgan failure, two cases of meningitis and abdominal pseudocyst formation related to the distal catheter tip and requiring catheter revision. These occurred at unclear time points and the patients were excluded from survival analysis by the study authors. However, the authors did report that at least one of the patients with meningitis and multiorgan failure died. Of the remaining patients, all survived to 12 months. No other complications or outcomes were reported in this group.

This study was classified as being globally weak with high risk of selection bias, a weak study design and small sample size which did not permit adjustment for factors potentially confounding outcome, and poorly documented exclusion from follow up causing high risk of attrition bias. Survival is a validated outcome measure but was unreliably reported in the group with complications.

#### Liu, et al. 2014 [72]

This retrospective single cohort study included nine patients with HIV-associated CM managed with VPS over a five-year period at a Shanghai hospital, China. Although exclusion criteria are documented, the number of exclusions is not. Included patients had a mean CD4+ cell count of 11 cells/μL. Neurosurgical intervention was considered in patients with raised lumbar puncture opening pressure and deteriorating neurological status despite antifungal therapy, repeated therapeutic lumbar puncture and mannitol administration. VPS was undertaken in all patients using a Medtronic® system, but the valve and catheter model are not documented. The authors define raised opening pressure as “> 400 cm H_2_0” which, presumably, is a typographic error (raised CSF opening pressure defined as > 20-35 cm H_2_O in other included studies [45, 53, 73–75]). Two patients were described as having loss of consciousness at baseline, but no objective measures were provided.

Eight of the nine patients studied were alive at 1 month. Three patients were lost to follow up at 6 months and no new deaths were reported. At 12 months, one more patient had died, yielding a survival of four out of six patients for whom follow-up was available. Given the low initial CD4 counts, it is likely that deaths would have occurred in those lost to follow-up by this time point and therefore there is a risk of attrition bias at this time point. The causes of death were VPS obstruction at 1 month and an unspecified reaction to antiretroviral drugs at 12 months. No other outcome measures, shunt failures or complications were reported.

This study was classified as being globally weak due to a weak study design and unknown risk of selection bias. No confounders were identified, and the primary outcome was survival – which is a robust outcome measure.

#### Vidal, et al. 2012 [53]

This retrospective cohort study investigated associations between various parameters derived from quantitative CSF microscopy with mortality in a cohort of patients who presented to hospital in São Paulo, Brazil, with HIV-associated CM. Included patients were admitted between January 2006 and June 2008, but it is unclear what rate of case acquisition this represents. A median CD4+ cell count of 36 cells/μL, with interquartile range of 17–87 cells/μL was reported across all patients. Neurosurgical intervention was considered in patients with lumbar puncture opening pressure persistently >20cmH_2_O after 14 days therapeutic lumbar puncture and antifungal treatment.

Although survival was presented in the series of 9 patients undergoing VPS, no other dependent or independent variables were reported in this subgroup to allow comparisons and therefore, for our purposes, this study provided case-series data. Initial neurological status and type of shunt valve or catheter were not described. Of the 9 patients who underwent VPS, 6 survived until hospital discharge. However, time to discharge and length of stay were not reported. No other outcomes were reported.

This study was classified as being globally weak with an unknown risk of selection bias and weak study design and size which did not permit adjustment for confounding variables regards our research question. Survival is a strong primary outcome measure.

### Included studies of tuberculous meningitis: characteristics and risk of bias

#### Nadvi, et al. 2000 [46]

This prospective cohort analysis compared outcome and mortality at 1 month following VPS between HIV-infected and HIV non-infected patients. Patients who underwent VPS for TBM, over an undefined recruitment period prior to commencement of the national South African antiretroviral treatment (ART) programme in 2004, were recruited from a single hospital in KwaZulu-Natal, South Africa. Of the 30 patients recruited, 15 were HIV-infected and 15 HIV non-infected. The two cohorts differed widely in age range with the HIV-infected group being mostly adult and the HIV non-infected group being mostly paediatric. Patients were diagnosed with hydrocephalus on the basis of CT, transcranial doppler or clinical observation in keeping with hydrocephalus and intraoperative ventricular pressure > 20cmCSF. Median initial GCS was 14 (range 9–15) in the HIV-infected group and 12 (range 9–15) in the HIV non-infected group. Use of EVD or therapeutic lumbar puncture was not documented for any patient. VPS was undertaken using a 102 cm catheter and low-profile, medium pressure Codman / Unishunt valve (Codman / Johnson & Johnson®, Raynham, MA).

At 1 month, 5/15 and 11/15 HIV-infected, and HIV non-infected patients were alive, respectively. 11/15 and 6/15 patients in the HIV-infected and HIV non-infected cohorts had an unfavourable Glasgow Outcome Score [43] (GOS 1–3) dichotomised GOS at 1 month. Multivariable analysis was not attempted. Univariate analysis revealed a significant association of CD4+ cell count and outcome (*p* = 0.031), but the magnitude of this association was not reported.

This study was classified as being globally weak. A prospective cohort study provides a moderate study design. The study included representative patients, but the control group was poorly age matched and this was not adjusted for. It is not clear that patients were enrolled consecutively, yielding an unknown risk of selection bias. The authors were not blinded to HIV status. Mortality is a robust primary outcome measure. As this study was undertaken prior to ART being made widely available in South Africa, its findings may not be generalisable to settings where ART is available [81].

#### Sharma, et al. 2015 [44]

This retrospective case-control study compared outcome and mortality between HIV-infected and HIV non-infected patients following VPS. All 30 eligible HIV-infected patients who underwent VPS at a single hospital in Bangalore, India, between June 2002 and October 2012 were included. These patients were compared against 30 HIV non-infected control patients that were matched for age, sex and clinical grade of TBM. Patients were included who “had symptoms of raised intracranial pressure, with clinical, radiological and CSF findings characteristic of TBM”. However, specific inclusion criteria were not documented. Initial GCS was dichotomized as 3–8 (unfavourable) and this was present in 13.3 and 3.3% in the HIV-infected and non-infected patients, respectively. The rest had GCS 9–15, which was classified as favourable. 23 and 22 patients in the HIV-infected and non-infected cohorts, respectively, had communicating hydrocephalus. Patients underwent VPS as a primary procedure unless extensive basal infarcts were evident on computed tomography (CT) scan of the brain. In these patients, a frontal EVD was placed and converted to VPS if improvement in “sensorium” was noted. The proportion of those who underwent primary VPS is not reported. VPS was with a medium-pressure Chhabra shunt (Surgiwear® Inc.). 9 HIV-infected patients and 4 HIV non-infected patients were lost to follow-up.

At 1 month, 13/21 HIV-infected and 20/26 HIV non-infected patients were alive. At 6 months, 9 HIV-infected and 18 HIV non-infected patients were alive. In the HIV-infected cohort, unfavourable initial GCS was associated with higher rates of mortality. GOS at 3 months was also dichotomized as unfavourable (1–3) or favourable (4, 5). 16/21 HIV-infected, and 9/26 HIV-non-infected patients had an unfavourable outcome at 3 months. Binomial logistic regression analysis confirmed that HIV infection (*p* = 0.038) and low Palur grade (*p* = 0.024) [82] were significantly associated with unfavourable outcome. No measure of effect was presented for these analyses. Anaemia (haemoglobin < 10 mg/dL) was associated with unfavourable outcome in the HIV-infected group (Exp.[β] = 25.6; *p* = 0.011).

This study was classified as being globally of moderate quality. A case-control study provides a moderate quality of study design. The study included representative consecutive cases with appropriate matching of controls. Potential confounders were adjusted for in the multivariable analysis. The authors were not blinded to HIV status. Just under a third of initially recruited patients were lost to follow-up at later time points, but this was appropriately documented. GCS and dichotomised GOS are robust outcome measures but not validated in the context of TBM. Mortality is a robust outcome measure.

#### Harrichandparsad, et al. 2019 [79]

This case control study compared 15 HIV-infected patients who were using ART prior to presentation who consecutively underwent VPS for TBM in 2017 with 15 historical retrospectively identified controls who underwent the same procedure but were not using ART. All patients receiving ART were prescribed single pill formulation of tenofovir and emtricitabine as well as efavirenz. The diagnosis of TBM was made on the basis of typical clinical, radiological and CSF findings. Not all patients had positive CSF cultures. All patients had radiological features of hydrocephalus with CSF pressures higher than 20 cm H_2_O on ventriculostomy and underwent VPS as a primary procedure. Both cohorts had similar initial GCS and Palur grade on admission.

All patients were followed up as inpatient for a minimum of 1 month post-operatively. Outcome was assessed at discharge using the dichotomised GOS (GOS 4–5 good outcome; 1–3 poor outcome). At discharge overall 15 patients achieved good outcome, one poor outcome and four died. Eleven patients receiving antiretroviral therapy achieved a good outcome with no instances of poor outcome and four deaths. This was better than the historical control group only four of which achieved a good outcome, one poor outcome and ten deaths. This difference was statistically significant in unadjusted analysis (*p* = 0.027). Patients in the ART treated group were more likely to have a higher GCS at 1 month than at presentation than the ART untreated group (*p* = 0.042).

This study was classified as being globally weak. A case-control study provides a moderate quality of study design. Consecutive patients were enrolled, reducing chances of selection bias and there was no attrition. Although matching of cases and controls by severity was attempted, differences in spread of age and gender were present. These were not statistically significantly different between groups at *p* < 0.05, but this may reflect high variation in these characteristics and the multivariable analysis that would be required to adjust for these was not possible. The study describes no blinding. GCS and dichotomised GOS are robust measures but not validated in the context of TBM. Mortality is a robust outcome measure.

#### Risk of bias between studies: tuberculous meningitis

As two comparative cohort studies were included assessing the effect of HIV infection on outcome, it was possible to pool reported measures of effect of HIV-infection on survival at one-month post-VPS. Risk of bias is summarised in Table 3.

**Table 3:**
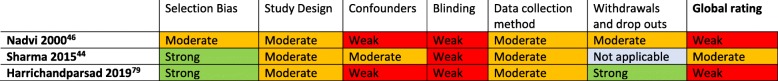
Quality assessment summary for tuberculous meningitis studies

Using the Canadian national collaborating center for methods and tools effective public health practice project Quality Assessment Tool for Quantitative Studies [49]

### Synthesis of results

#### Effect of pathology

Nine studies of survival and/or outcome in TBM and CM were identified [44–46, 53, 72–75, 79]. However, none of these compared survival or outcome between TBM and CM. Study populations, outcome measures and follow-up varied greatly. Further, all of the studies of CM were of weak design, and two of the three studies of TBM also being graded as weak. There was significant methodological heterogeneity between CM and TBM studies and it was therefore not possible to conduct meaningful meta-analytic comparison of outcomes between these groups. We therefore present a narrative analysis. The results of pooling of reported outcomes within each group are presented in SOF tables, with the purpose of summarising reported outcomes to date. These are of very low to low GRADE of evidence (see risk of bias, above), indicating that the true outcomes for each pathology may be, or are likely to be, substantially different from the pooled measure [83]..

### Cryptococcal meningitis

Pooled outcome measures are reported in Table 4.

**Table 4:** Summary of findings – cryptococcal meningitis

Outcomes	Pooled outcome	№ of participants (studies)	Quality of the evidence (GRADE)	Comments
Survival				
Survival follow up: 1 months	17 (81.0%; range 62.5–100%)	21 (3 observational studies) [45, 72, 74]	⨁◯◯◯ VERY LOW ^a,b,d,f,g,h,k^	
Survival follow up: 6 months	16 (76.2%; range 62.5–100%)	21 (4 observational studies) [45, 72, 74, 75]	⨁◯◯◯ VERY LOW ^a,b,d,f,g,h^	
Survival follow up: 12 months	20 (69.0%; range 33–100%)	29 (4 observational studies) [45, 72–74]	⨁◯◯◯ VERY LOW ^a,b,d,f,g,h,i^	
AIDS specific mortality				
AIDS specific mortality follow up: 1 months	1 (4.8%; range 0–12.5%)	21 (3 observational studies) [45, 72, 74]	⨁◯◯◯ VERY LOW ^a,b,d,f,g,h,k^	
AIDS specific mortality follow up: 6 months	3 (16.7%; range 12.5–33%)	18 (3 observational studies) [45, 72, 74]	⨁◯◯◯ VERY LOW ^a,b,d,f,g,h,k^	
AIDS specific mortality follow up: 12 months	5 (29.4%; range 25–66%)	17 (3 observational studies) [45, 72, 74]	⨁◯◯◯ VERY LOW ^a,b,d,f,g,h,k^	
VPS survival				
VPS survival follow up: 1 months	8 (88.9%)	9 (1 observational study) [72]	⨁◯◯◯ VERY LOW ^a,d,e,f,g^	Cherian, et al. [74] reported two shunt failures at unspecified timepoints.
				Corti, et al. [73] reported three shunt failures at unspecified timepoints.
VPS survival follow up: 6 months	5 (83.3%)	6 (1 observational study) [72]	⨁◯◯◯ VERY LOW ^a,d,e,f,g^	Cherian, et al. [74] reported two shunt failures at unspecified timepoints.
				Corti, et al. [73] reported three shunt failures at unspecified timepoints.
VPS survival follow up: 12 months	5 (83.3%)	6 (1 observational study) [72]	⨁◯◯◯ VERY LOW ^a,d,e,f,g^	Cherian, et al. [74] reported two shunt failures at unspecified timepoints and one at 12 months.
				Corti, et al. [73] reported three shunt failures at unspecified timepoints.
Operative complication	4 (23.5%; range 0–50%	17 (2 observational studies) [72, 74]	⨁◯◯◯ VERY LOW ^a,b,c,d,e,f,g^	Corti,et al. [73] reported one case of multiorgan failure or unspecified aetiology.
Validated outcome score	No studies reported validated outcome measures at any time point			Calvo, et al. [75] reported unvalidated outcome measures at 6 months

a. Possibly selected observational case series

b. Population includes some HIV non-infected individuals

c. Outcomes reported at different time points for different patients

d. Population derived from a study indirectly addressing question

e. Outcomes not fully defined

f. CD4 count not described or unusually low in some studies

g. Population baseline characteristics described incompletely

h. Significant loss to follow up

i. Exclusion of patients who suffered postoperative complications

k. Loss of case to follow up at unspecified time point

#### Survival

Three studies reported survival in CM at one-month post VPS [45, 72, 74]. Cherian, et al. reported three deaths in eight patients. Three of these patients had a diagnosis of CM IRIS, one of which died (AIDS-specific mortality). Liu, et al. report one death in nine patients at 1 month. This was due to shunt obstruction. Bach, et al.’s four patients with documented follow up were all alive at 1 month. In total, 17 of 21 (81%) patients described in these studies survived 1 month, with one episode of shunt failure documented.

At 6 months post-VPS, four studies reported survival [45, 72, 74, 75]. Three of Liu, et al’s patients had been lost to follow-up. However, no new deaths were confirmed. Bach, et al. reported one death due to HIV-wasting syndrome. Another had died of cytomegalovirus pancreatitis. These both therefore contributed to AIDS-specific mortality [84]. Calvo, et al. obtained 3 months follow up for three patients, all of whom survived. Cherian et al. followed up all of their eight patients and reported no new deaths. With loss to follow-up and addition of three surviving patients from Calvo, et al’s cohort, overall survival remained stable: 17 of 21 (81%) patients that were followed up at 6 months were alive. No new cases of shunt failure were documented.

Four studies reported 12-month survival [45, 72–74]. Liu, et al. reported a further death. Cherian, et al. reported a death in one of their patients who had been diagnosed with CM IRIS, contributing to AIDS-specific mortality. Another of their patients was lost to follow-up. One of Bach et al.’s patients had died of *Pneumocycstis jiroveci* pneumonia, also contributing to AIDS-specific mortality. Corti, et al. report that all of their 12 patients followed-up for 1 year were alive. In total 20 of 29 patients with 12-month follow-up survived. Five of the 17 (29%) patients reported by studies describing cause of death had AIDS-defining illness as a primary or major contributing cause of death by 12 months.

Liu et al., reported one shunt failure at 1 month. Cherian and colleagues reported one shunt failure at 12 months, but as they also reported two other instances of shunt failure at unclear timepoints, their shunt failure data was not included in the pooled analysis.

#### Operative complications

Complications of treatment were reported by two studies [72, 74]. In Liu, et al.’s series, none of these were related to the surgical management of their patients at 12 months. In addition to their three instances of shunt failure, Cherian and colleagues reported one instance of over drainage subdural haematoma at an unspecified time. Follow-up in this series ranged from 28 to 1512 days.

#### Functional outcome

No studies reported any measures of functional outcome at any time point. Calvo, et al. reported that two of three patients made a “good recovery” at 6 months but this measure is not validated [75].

### Tuberculous meningitis

Pooled outcome measures are reported in Table 5.

**Table 5:** Summary of findings – tuberculous meningitis

Outcomes	Pooled outcome	№ of participants (studies)	Quality of the evidence (GRADE)	Comments
Survival				
Survival follow up: 1 months	34 (51.5%; range 33.3–61.9%)	66 (3 observational studies) [44, 46, 79]	⨁⨁◯◯ LOW ^a^	
Survival follow up: 6 months	9 (42.9%)	21 (1 observational study) [44]	⨁◯◯◯ VERY LOW ^a,b^	
Survival follow up: 12 months	7 (33.3%)	21 (1 observational study) [44]	⨁◯◯◯ VERY LOW ^a,b^	
AIDS specific mortality	No studies reported AIDS specific mortality at any point			
VPS survival	No studies reported VPS failure or survival rates at any time point			
Operative complication	No studies reported rates of operative complication at any time point			
Validated outcome score				
Glasgow outcome score (GOS) follow up: 1 month	GOS 1–3: 26 (57.8%) GOS 4–5: 19 (42.2%)	45 (2 observational studies) [46, 79]	⨁⨁◯◯ LOW ^a^	GOS 1–3 signifies death to severe disability GOS 4–5 signifies moderate to low disability
Glasgow outcome score (GOS) follow up: 6 months	GOS 1–3: 16 (76.2%) GOS 4–5: 5 (23.8%)	21 (1 observational study) [46]	⨁◯◯◯ VERY LOW ^a,c^	GOS 1–3 signifies death to severe disability GOS 4–5 signifies moderate to low disability
Glasgow outcome score (GOS) follow up: 12 months	No studies reported a validated outcome score at 12 months			

a Possibly selected observational case series level data

b Incomplete long-term follow-up

c Mean follow up 130 days. Range of 3–25 months

#### Survival

Three studies reported 1-month survival following VPS for HIV-associated TBM [44, 46, 79]. Sharma et al. reported 13 of 21 patients surviving for one-month post-VPS. Nadvi, et al. reported just 5 of 15 patients surviving for one-month. Harrichandparsad, et al. reported 14 out of 30 patients surviving for 1 month, although 10 of these deaths were in the ART untreated group.

Sharma et al. reported 9 of 21 patients surviving 6 months. Sharma et al. also reported “long-term” survival of 7 of 21 with mean follow-up of 130 days, range 91–760 days. No studies reported causes of death or measures of shunt survival.

#### Operative complications

No studies reported rates of operative complication.

#### Functional outcome

Two studies reported GOS in HIV-infected patients 1 month following VPS [46, 79]. 11 of 15 patients in Nadvi, et al.’s study had an unfavourable functional outcome, with 10 of these being dead. Therefore four of the five surviving patients attained GOS 4–5, signifying moderate to low levels of disability at one month [43]. Harrichandparsad, et al. reported 15 of 30 patients achieving an unfavourable outcome, including death at one month, 11 of which were in the ART untreated group [79].

### Effect of human immunodeficiency virus infection

As two studies comparing HIV-infected and HIV non-infected patients with TBM were identified, it was possible to examine the impact of HIV-infection on survival [44, 46].

**Fig. 2:**
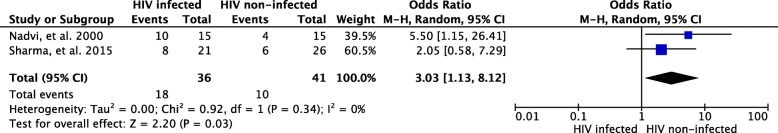
Forest Plot - One-month mortality HIV-infected vs. HIV non-infected tuberculous meningitis

At 1 month, the odds ratio for mortality in Sharma, et al.’s HIV-infected group was 2.02 (95% confidence interval [CI]: 0.58–7.01). In Nadvi’s group it was 4.73 (95% CI: 0.58–7.01). The pooled odds ratio (Mantel-Haenszel) for one-month mortality in the HIV-infected group was 3.03 (95% CI: 1.13–8.12; *p* = 0.03; chi^2^ = 0.92; df = 2; I^2^ = 0%; Fig. 2), indicating significantly increased risk of mortality at one-month post-VPS in HIV infection.

Sharma et al. reported six-month survival, but Nadvi, et al. did not.

## Discussion

### Summary of evidence

Only studies of patients with who underwent ventriculoperitoneal shunting because of HIV-associated hydrocephalus secondary to TBM or CM were identified by our study. Six series of patients with CM [45, 53, 72–75] and two controlled studies of patients with TBM [44, 46] were included.

#### Cryptococcal meningitis

The included studies of CM provided a very low level of evidence on which to inform clinical practice. Reported survival by included studies of CM varied extremely widely – from 33 to 100% at 1 year – and this is likely a consequence of study heterogeneity. The relatively high pooled 12-month survival of 69% might represent selection and attrition biases, which many included studies were considered to be at a high risk of. For comparison, 12-month survival in a study of 549 patients with HIV-associated CM who did not receive shunting varies from 55 to 93%, contingent on patients’ antiretroviral status [85].

The 2018 World Health Organisation (WHO) guidelines on CM recommend that CM-associated hydrocephalus be initially managed by therapeutic lumbar puncture and drainage of CSF [86]. These guidelines emphasise that repeat lumbar puncture should be undertaken to control symptomatic hydrocephalus until resolution and do not describe any role for permanent CSF diversion in CM. The Infectious Diseases Society of America 2010 guidelines for the management of cryptococcal disease recommended early ventricular CSF diversion for non-communicating hydrocephalus in CM [87]. For chronic communicating hydrocephalus or communicating hydrocephalus causing significant neurological impairment, permanent CSF diversion may be undertaken [87].

#### Tuberculous meningitis

Three cohort studies were included. These provide a low level of evidence to inform prognostication at one-month, in terms of survival and functional outcome, for patients undergoing VPS for HIV-associated TBM. One-month survival varied from 33 to 61.9% between these studies, yielding a very cautious pooled estimate of one-month survival of 51.5% and a pooled estimate of 42.2% good outcome at 1 month. We anticipate that this estimate differs substantially from the true outcome. The included studies provide a very low level of evidence to inform later prognostication in terms of survival and functional outcome. For all other measures of outcome – including AIDS-specific mortality, shunt failure risk and perioperative complication – the included studies provided no data to inform clinical decision-making. Nadvi, et al. and Sharma, et al’s studies demonstrate that patients who underwent VPS for HIV-associated TBM had poorer outcomes than their HIV non-infected counterparts. Meta-analysis of these findings yielded increased risk of mortality (odds ratio 2.93) in the HIV-infected group. However, one of these studies compared a largely paediatric HIV non-infected population with a largely adult HIV-infected population [46]. The clinical phenotypes and pathophysiological responses to brain injury in these groups differ widely and so comparisons should be interpreted with caution [88, 89]. It is also important to note that whilst one of the studies employed ventricular CSF diversion as a primary measure for management of hydrocephalus [44], it is unclear if this was undertaken primarily, or following failure of lumbar drainage/medical management in the other study [46]. This heterogeneity limits the interpretation of comparisons between reported outcomes in HIV-infected and non-infected cohorts.

The British Infection Society published guidelines in 2009 for the management of TBM [90]. These guidelines did not recommend altering the medical management of patients with TBM on the basis of their HIV status but did not comment on their surgical management with respect to HIV. On the basis of observational studies of HIV non-infected patients, early VPS is recommended by these guidelines for non-communicating hydrocephalus [90–92]. It is noted that response to external ventricular drainage poorly predicts response to VPS [91]. Our study’s findings would indicate that HIV status might impact significantly on surgical outcome and therefore caution should be employed by future guideline working groups before generalizing the results of studies conducted of HIV non-infected populations to inform the surgical management of HIV-infected patients.

### Review limitations

This review is limited in certain respects. As just two studies reporting effect sizes could be included in our meta-analysis, it was not possible to meaningfully assess for publication bias. Publication bias in case series tends to over-represent good outcomes and therefore our pooled results are likely to indicate better survival and outcomes than is the truly the case [93]. Included studies in our review were of low-moderate quality study design. The decision to utilise designs such as single cohort studes or small case series is often a pragmatic choice on behalf of the study authors: conduct of larger standardised observational studies, with blinding of outcome assessment and multivariable adjustment of factors influencing clinical outcome may not have been justified by available resources. Unfortunately, observer bias and the effect of unmeasured confounding factors in these types of study can substantially impact on study outcome and this therefore reduces our confidence in our pooled estimates of outcome following VPS insertion in HIV-infected individuals [94, 95].

Four of our included studies were published 1997–2003, prior to the widespread availability of ART [81]. Use of ART is an important predictor of survival and this could potentially impact on the generalisability of these studies outcomes to contemporary practice [86, 90]. These included Nadvi et al’s study of TBM, which reported lower one-month survival (at 33%) than the more recent study by Sharma, et al. (61.9%) [44, 46, 81]. Harrichandparsad, et al. directly assessed the impact of ART provision and showed a dramatic, yet expected, association between ART provision and better outcomes in the context of TBM (ART 73% vs. no ART 33% 1 month survival) [79]. It is striking that 1 month survival in this small ART treated cohort is similar to that of the pooled data from HIV uninfected patients in Sharma et al., and Nadvi, et al’s studies (75.6%). Two studies of CM published in 2003 [75] and 1997 [45] reported 0–50% 6 month mortality, respectively. However, these contributed small numbers of cases to our analysis of CM and so the effect of lack of availability of ART for these cases on our analysis is likely less than the effect of other factors such as incomplete follow-up.

### Suggestions for future research

Our review has included studies of patients with either TBM or CM. Although other causes of hydrocephalus in HIV have been reported, none of these reports met our criteria for inclusion [5–8, 96, 97]. Understanding how outcomes differ following VPS insertion in HIV-infected and non-infected patients with causes of hydrocephalus not directly attributable to HIV infection (e.g. subarachnoid haemorrhage, Chiari malformation, etc) would allow determination of the relative impacts of undertaking VPS in the context of active CSF infection and HIV-infection. Unfortunately, to date, most large studies of VPS insertion for “all cause” hydrocephalus have excluded HIV-infected patients, have been conducted in populations with low HIV-infection prevalence, or do not report HIV-infection rates within their cohort [30, 98, 99].

Furthermore, our review has provided evidence that available data is of inadequate quality to reliably inform clinical decision-making related to VPS insertion for HIV-associated CM and TBM. The burden of HIV-associated CM and TBM in low-middle income settings in the era of ART are unclear yet believed to remain significant, with one study estimating over 600,000 HIV-associated CM deaths occurring in 2006 [37] and TBM and CM accounting for approximately 10% of AIDS related deaths in a Ghanaian study from 2007 [36]. As hydrocephalus or raised CSF pressure is present in approximately 50% of cases of HIV-associated TBM [100, 101] and 60% of HIV-associated CM [102], the lack of evidence to guide use of VPS in this setting is concerning.

We therefore propose that a large, multi-centre, prospective, population-based survival analysis of all patients with HIV-infection and hydrocephalus be urgently conducted. This should include both surgically and non-surgically managed patients with hydrocephalus occurring in association of HIV, either as a consequence of HIV-infection or any other cause, not directly related to HIV-infection. Such a study should collect baseline clinical data – such as pre-admission ART use, neurological grade, CD4+ cell count, haemoglobin and CSF cell count, protein and glucose, microbiological results and opening pressure – which are currently used to prognosticate and guide selection of surgical candidates. Ideally, all included patients would be matched to HIV non-infected controls, however identification of appropriate control subjects might be difficult for conditions such as CM, which infrequently affect HIV non-infected individuals [103]. This study would allow validation of these potential markers of prognosis and would allow identification of patient groups for which there may be equipoise regarding the benefit and harms of VPS. Definition of such populations would provide a strong rationale for undertaking an appropriately powered randomised clinical trial, conducted in low-middle income settings.

## Conclusions

It is not possible to reliably determine the risks and benefits of VPS surgery in patients with HIV on the basis of the currently available literature. All studies of outcomes following VPS for HIV-associated CM are weak, with only case-series data available. Studies of survival and/or outcome following VPS for HIV-associated CM and TBM are either weak or of moderate quality. This included three cohort studies of patients with TBM, at differing risk of bias. Included studies indicate that only a very low to low quality evidence base exists on which to inform clinical decision making regarding VPS insertion for HIV-infected patients. This is reflected in weak recommendations in existing clinical guidelines for neurosurgical practice in HIV-infected patients [87, 90, 104, 105]. In the first instance, a rigorously conducted prospective, population based observational study of outcome and survival in HIV-associated hydrocephalus should be conducted. This is an urgent research priority and would inform design of future randomised clinical trials.

## Supplementary information

**Additional file 1.** Supplementary Material: search terms

## Data Availability

All study data and search strategy outputs will be made available on reasonable request to the corresponding author.

## Abbreviations

HIV: Human immunodeficiency virus
AIDS: Acquired immune deficiency syndrome
CSF: Cerebrospinal fluid
CNS: Central nervous system
TBM: Tuberculous meningitis
CM: Cryptococcal meningitis
IRIS: Immune reconstitution inflammatory syndrome
ART: Antiretroviral therapy
VPS: Ventriculoperitoneal shunt
EVD: External ventricular drain
GCS: Glasgow coma score
ICU: Intensive care unit
GOS: Glasgow outcome score
CT: Computed tomography
