# Supplementary material for: Ventriculoperitoneal shunt insertion in human immunodeficiency virus infected adults: a systematic review and meta-analysis
Source: BMC Neurol. 2020 Apr 17;20:141. doi: 10.1186/s12883-020-01713-4 (PMC7164262; doi:10.1186/s12883-020-01713-4)
Supplement: Supplementary file 1 — Additional file 1. Supplementary Material: search terms [file 12883_2020_1713_MOESM1_ESM.docx]

# Supplementary Material: search terms

## MEDLINE (PubMed)

**Abbreviated search strategy (full syntax available on request)**

1. human
2. immunodeficiency
3. virus
4. 1 AND 2 AND 3
5. human immunodeficiency virus
6. HIV
7. acquired
8. immunodeficiency
9. 7 AND 8
10. acquired immunodeficiency syndrome
11. AIDS
12. 4 OR 5 OR 6 OR 9 OR 10 OR 11
13. ventriculoperitoneal
14. ventric*
15. ventricular
16. 14 OR 15
17. peritone*
18. peritoneal
19. 17 OR 18
20. 16 AND 19
21. VP
22. cerebrospinal
23. cerebrosp*
24. CSF
25. 22 OR 23 OR 24
26. 13 OR 20 OR 21 OR 25
27. shunt*
28. shunt
29. catheter*
30. catheter
31. diversion
32. divers*
33. 27 OR 28 OR 29 OR 30 OR 31 OR 32
34. 26 AND 33
35. ventriculoperitoneal shunt
36. 34 OR 35
37. 12 AND 36

## CENTRAL

1. Human
2. Immunodeficiency
3. Virus
4. 1 and 2 and 3
5. MeSH descriptor: [HIV] explode all trees
6. Human immunodeficiency virus
7. HIV
8. acquired
9. Immunodeficiency
10. 8 and 9
11. acquired immunodeficiency syndrome
12. AIDS
13. MeSH descriptor: [Acquired Immunodeficiency Syndrome] explode all trees
14. #4 or #5 or #6 or #7 or #10 or #11 or #12 or #13
15. ventriculoperitoneal
16. ventricular
17. peritoneal
18. 16 and 17
19. VP
20. cerebrospinal
21. CSF
22. MeSH descriptor: [Cerebrospinal Fluid] explode all trees
23. 15 or 18 or 19 or 20 or 21 or 22
24. shunt
25. MeSH descriptor: [Cerebrospinal Fluid Shunts] explode all trees
26. catheter
27. MeSH descriptor: [Catheters] explode all trees
28. diversion
29. #24 or #25 or #26 or #27 or #28
30. 23 and 29
31. ventriculoperitoneal shunt
32. MeSH descriptor: [Ventriculoperitoneal Shunt] explode all trees
33. 30 or 31 or 32
34. 14 and 33

## EMBASE

Unlimited search terms linked to Subject Heading

1. human/
2. immunodeficiency.mp. or immune deficiency/
3. virus/
4. 1 and 2 and 3
5. human immunodeficiency virus.mp. or Human immunodeficiency virus/
6. HIV.mp. or Human immunodeficiency virus/
7. acquired.mp.
8. immunodeficiency.mp. or immune deficiency/
9. 7 and 8
10. acquired immunodeficiency syndrome.mp. or acquired immune deficiency syndrome/
11. AIDS.mp. or acquired immune deficiency syndrome/
12. 4 or 5 or 6 or 9 or 10 or 11
13. cerebrospinal fluid shunting/ or shunting/ or shunt infection/ or hydrocephalus/ or brain ventricle peritoneum shunt/ or ventriculoperitoneal.mp. or cerebrospinal fluid/
14. ventricular.mp
15. brain ventricle peritoneum shunt/ or hydrocephalus/ or ventriculoatrial shunt/ or ventric*.mp.
16. 14 or 15
17. peritoneum/ or peritone*.mp.
18. peritoneal catheter/ or peritoneal drain/ or peritoneal.mp. or peritoneal cavity/
19. 17 or 18
20. 16 and 19
21. vp.mp.
22. cerebrospinal fluid level/ or cerebrospinal.mp. or cerebrospinal fluid drainage system/ or cerebrospinal fluid analysis/ or cerebrospinal fluid/ or cerebrospinal fluid shunting/ or cerebrospinal fluid drainage/
23. cerebrospinal fluid/ or cerebrosp*.mp
24. CSF.mp. or cerebrospinal fluid/
25. 22 or 23 or 24
26. 13 or 20 or 21 or 25
27. brain ventricle peritoneum shunt/ or shunt.mp. or shunt occlusion/ or ventriculoatrial shunt/ or shunt thrombosis/ or shunt failure/ or shunt infection/
28. shunt*.mp.
29. catheter.mp. or catheter fracture/ or intrathecal catheter/ or drainage catheter/ or catheter infection/ or neurological catheter/ or catheter care/ or catheter leakage/ or subdural catheter/ or peritoneal catheter/ or catheter occlusion/ or catheter sheath/ or catheter thrombosis/ or ventriculostomy catheter/ or catheter/ or catheter dislocation/ or antimicrobial catheter/ or catheter migration/ or catheter complication/ or catheter valve/ or intracranial catheter/
30. catheter/
31. diversion.mp.
32. divers*.mp.
33. 27 or 28 or 29 or 30 or 31 or 32
34. 26 and 33
35. ventriculoperitoneal shunt.mp. or brain ventricle peritoneum shunt/
36. 34 or 35
37. 12 and 36

## CINAHL Plus (EBSCOhost)

S1. MW Human immunodeficiency virus

S2. TI Human AND TI Immunodeficiency AND TI virus

S3. (MH "HIV-Infected Patients") OR (MH "HIV Protease Inhibitors") OR (MH "HIV Seropositivity") OR (MH "AIDS Serodiagnosis")

S4. TX acquired OR TX immunodeficiency

S5. MW aquired immunodeficiency syndrome OR TX AIDS

S6. S1 OR S2 OR S3 OR S4 OR S5

S7. TX ventric* OR TX ventricular

S8. TX peritoneal AND TX peritone*

S9. S7 AND S8

S10. TX ventriculoperitoneal

S11. TX VP

S12. TX cerebrospinal OR MW cerebrospinal OR TX cerebrosp*

S13. TX CSF

S14. S12 OR S13

S15. S9 OR S10 OR S11 OR S14

S16. TX shunt* OR MW shunt

S17. TX catheter* OR MW catheter

S18. MW diversion OR TX divers*

S19. S16 OR S17 OR S18

S20. S15 AND S19

S21. TX ventriculoperitoneal shunt OR MW ventriculoperitoneal shunt

S22. S20 OR S21

S23. S6 AND S22

## LILACS (VHL)

((((human) AND (immunodeficiency) AND (virus)) OR (human immunodeficiency virus) OR (HIV) OR ((acquired) AND (immunodeficiency)) OR (acquired immunodeficiency syndrome) OR (AIDS))) AND (((ventriculoperitoneal) OR (((ventric*) OR (ventricular)) AND ((peritone*) OR (peritoneal))) OR (VP) OR ((cerebrospinal) OR (cerebrosp*) OR (CSF)))) AND ((shunt*) OR (shunt) OR (catheter*) OR (catheter) OR (diversion) OR (divers*) OR (ventriculoperitoneal shunt))

## Research Registry (www.researchregistry.com)

Individual searches for:

1. HIV
2. AIDS
3. acquired immunodeficiency syndrome
4. Human immunodeficiency
5. ventriculoperitoneal
6. hydrocephalus
7. cerebrospinal
8. CSF
9. shunt

## The metaRegister of Controlled Trials (mRCT) (www.controlled-trials.com)

((((human) AND (immunodeficiency) AND (virus)) OR (human immunodeficiency virus) OR (HIV) OR ((acquired) AND (immunodeficiency)) OR (acquired immunodeficiency syndrome) OR (AIDS))) AND (((ventriculoperitoneal) OR (((ventric*) OR (ventricular)) AND ((peritone*) OR (peritoneal))) OR (VP) OR ((cerebrospinal) OR (cerebrosp*) OR (CSF)))) AND ((shunt*) OR (shunt) OR (catheter*) OR (catheter) OR (diversion) OR (divers*) OR (ventriculoperitoneal shunt))

## ClinicalTrials.gov (www.clinicaltrials.gov)

(HIV OR (Human immunodeficiency virus) OR AIDS) AND ((Ventriculoperitoneal Shunt) OR (CSF diversion) OR Hydrocephalus)

## OpenGREY

((((human) AND (immunodeficiency) AND (virus)) OR (human immunodeficiency virus) OR (HIV) OR ((acquired) AND (immunodeficiency)) OR (acquired immunodeficiency syndrome) OR (AIDS))) AND (((ventriculoperitoneal) OR (((ventric*) OR (ventricular)) AND ((peritone*) OR (peritoneal))) OR (VP) OR ((cerebrospinal) OR (cerebrosp*) OR (CSF)))) AND ((shunt*) OR (shunt) OR (catheter*) OR (catheter) OR (diversion) OR (divers*) OR (ventriculoperitoneal shunt))

## African Journals Online (AJOL)

(((human immunodeficiency virus) OR (acquired immunodeficiency syndrome) OR (AIDS))) AND (((ventriculoperitoneal) OR (((ventric*) OR (VP) OR ((cerebrosp*) OR (CSF)))) AND ((shunt*) OR (catheter*) OR (divers*) OR (ventriculoperitoneal shunt))

# Supplementary material: data extraction proforma

|  | **Data to be extracted** | **Data** |
| --- | --- | --- |
| Population | Age range |  |
|  | Diagnostic modality for hydrocephalus/CSF hypertension |  |
|  | Diagnostic modality for underlying aetiology |  |
|  | CD4+ cell count range (for each underlying aetiology) |  |
|  | Country population derived from |  |
|  | Severity of disease at baseline (using GCS, palur grade, MRC scale, GOS or mRS) for each underlying aetiology |  |
|  | Communicating or non-communicating hydrocephalus |  |
| Intervention | Primary VPS or following EVD |  |
|  | Catheter type (plain silicon, antibiotic impregnated, silver impregnated) |  |
| Comparison intervention | Description of intervention |  |
|  | Frequency of intervention |  |
| Outcome (for each underlying aetiology and intervention) | Survival at one month, six months and twelve months. |  |
|  | Causes of death at one month, six months and twelve months. |  |
|  | Rate of shunt failure at one month, six months and twelve months. |  |
|  | Rates of complication at one month, six months and twelve month. |  |
|  | Validated outcome measure at one month, six months, and twelve months. |  |
